# MiR-199a-3p/5p participated in TGF-β and EGF induced EMT by targeting DUSP5/MAP3K11 in pterygium

**DOI:** 10.1186/s12967-020-02499-2

**Published:** 2020-09-01

**Authors:** Siying He, Yifang Huang, Shiqi Dong, Chen Qiao, Guohua Yang, Shuai Zhang, Chen Wang, Yuting Xu, Fang Zheng, Ming Yan

**Affiliations:** 1 Center for Gene Diagnosis, and Clinical Laboratory, Zhongnan Hospital of Wuhan University, Donghu Rd 169#, Wuhan, 430071 China; 2 Department of Clinical Laboratory, the First Affiliated Hospital of Guangxi Medical University, Nanning, 530021 Guangxi China; 3 Department of Ophthalmology, Zhongnan Hospital of Wuhan University, Wuhan, 430071 Hubei China; 4 Department of Corneal, Hankou Aier Eye Hospital, Wuhan, 430024 Hubei China; 5 Demonstration Center for Experimental Basic Medicine Education, Wuhan University, Wuhan, 430071 Hubei China

**Keywords:** miR-199a, *DUSP5*, *MAP3K11*, Pterygium, EMT

## Abstract

**Background:**

Recently, it has been reported that miRNA is involved in pterygium, however the exact underlying mechanism in pterygium is unrevealed and require further investigation.

**Methods:**

The differential expression of miRNA in pterygium was profiled using microarray and validated with quantitative real-time polymerase chain reaction (qRT-PCR). Human conjunctival epithelial cells (HCEs) were cultured and treated with transforming growth factor β (TGF-β) and epidermal growth factor (EGF) and transfected with miR-199a-3p/5p mimic and inhibitor. Markers of epithelial-mesenchymal transition (EMT) in HCEs were detected using western blot and immunohistochemistry. Cell migration ability was determined using wound healing and transwell assay, while apoptosis was determined by flow cytometry. The target genes of miR-199a were confirmed by the dual-luciferase reporter assay.

**Results:**

TGF-β and EGF could induced EMT in HCEs and increase miR-199a-3p/5p but suppress target genes, DUSP5 and MAP3K11. With the occurrence of EMT, cell migration ability was enhanced, and apoptosis was impeded. Promoting miR-199a-3p/5p expression could induce EMT in HCEs without TGF-β and EGF, while suppressing miR-199a-3p/5p could inhibit EMT in TGF-β and EGF induced HCEs. In a word, TGF-β and EGF induced EMT could be regulated with miR-199a-3p/5p-DUSP5/MAP3K11 axes. The validated results in tissues showed that, compared with control conjunctival tissues, miR-199a-3p/5p were more overexpressed in pterygium, while DUSP5/MAP3K11 were lower expressed. In addition, bioinformatics analysis indicated the miR-199a-3p/5p-DUSP5/MAP3K11 was belong to MAPK signalling pathway.

**Conclusions:**

TGF-β and EGF induce EMT of HCEs through miR-199a-3p/5p-DUSP5/MAP3K11 axes, which explains the pathogenesis of EMT in pterygium and may provide new targets for pterygium prevention and therapy.

## Background

Pterygium is a common ocular surface disease with a triangular-shaped lesion growing in the limbal conjunctiva and progressively invading toward cornea, affecting nearly 200 million people globally and the prevalence can even be as high as 22% in some countries [1–4]. Ultraviolet light (UVB) exposure induced chronic irritation of eyes is widely considered as the dominant risk factor of pterygium. Excessive UVB can lead to the inactivation of p53, and inactivated p53 promoted the process of epithelial-mesenchymal transition (EMT), contributing to the pathogenesis of pterygium [5–7]. Nowadays, conjunctival auto-transplantation is the most common treatment for pterygium, but the recurrence rates range from 2 to 39% [8–10].

The process of EMT, manifested in epithelial cells losing the polarity and cell–cell adhesion, and obtaining the migratory ability to become mesenchymal cells, has been verified as one of the most significant characteristic of pterygium [11–14]. Transforming growth factor β (TGF-β and epidermal growth factor (EGF) were found to be two fatal inducement in the process of EMT in various diseases [14–17]. TGF-β and EGF are overexpressed in pterygium, and it has been reported that both of them can be activated by UVB [18, 19]. TGF-β signalling stimulates fibroblasts migration, proliferation and myofibroblasts differentiation, which play a significant role as profibrotic agents in pterygium [20, 21]. While EGF promotes excessive keratinization and the overexpressed receptor of EGF (EGFR) leaded to increased proliferation in pterygium epithelial cells [22, 23]. So, we planned to induce EMT in human conjunctival epithelial cells (HCEs) with TGF-β and EGF, which imitates the EMT initiation in pterygium as a cell model.

On another hand, current studies indicated miRNAs were involved in pterygium [2, 6, 24–27], but based on a are a relatively small number of samples [2, 26–28]. MiRNAs are a large class of small non-coding RNAs, which can complementarily or partially complementarily bind to the 3′-untranslated regions (3′-UTR) of target mRNAs, leading to degradation or translation repression [29, 30]. Based on results of miRNA microarray in the present study, miR-199a-3p/5p showed a significantly higher expression level in pterygium that in conjunctiva. MiR-199a-3p/5p was reported to suppress EMT in various cancer, such as testicular germ cell tumor, non-small cell lung cancer, head and neck cancer, and so on [31–33]. But they were also reported to promote the EMT process in hepatic fibrosis and idiopathic pulmonary fibrosis (IPF) [15, 34, 35]. These contradictory results suggest that miR-199a-3p/5p might have different functions in different types of EMT in various diseases. Pterygium is essentially characterized as a kind of fibrosis disease, since after EMT, the conjunctival epithelial cells obtain motility and grow toward the cornea, rather than inter-tissue invasion like tumor.

So, we hypothesized that miR-199a-3p/5p might promote EMT in fibrotic pterygium. Our objective was to explore the role of miR-199a-3p/5p in EMT of pterygium based on the established pterygium EMT cell model using HCEs with TGF-β and EGF, and eventually to find new perspectives on treatments for pterygium occurrence and development.

## Material and methods

### Pterygium samples and control conjunctiva tissue samples

A total of 263 conjunctiva tissues were analysed in this study, including 234 pterygium samples and 29 control conjunctiva tissues. All pterygium samples were obtained from patients who diagnosed with primary pterygium undergoing a surgical resection and control conjunctiva tissues were collected from patients who underwent cataract surgery from December 2014 to October 2019 at Zhongnan Hospital of Wuhan University. Samples were stored at -80 °C immediately after surgery until used.

This study was approved by the Medical Ethical Committee of Zhongnan Hospital of Wuhan University and followed the tenets of the Declaration of Helsinki and its later amendments. Informed consents were obtained from all patients before the study was carried out.

### MiRNA microarray assay

Total RNA was isolated from frozen tissue using miRNeasy Mini Kit (Qiagen, Hilden, Germany) according to the manufacturer’s instruction before evaluated by the NanoDrop2000 spectrophotometer (Thermo Scientific, MA, USA). Total RNA was purified and labelled using FlashTag™ Biotin HSR RNA Labelling Kit (P/N 901,911, Affymetrix) according to the manufacturer’s instructions to obtain biotin labelled miRNA. Array hybridization and wash were performed by GeneChip® Hybridization, Wash and Stain Kit (P/N900720, Affymetrix Santa Clara CA, USA) and GeneChip Eukaryotic Hybridization Control Kit (P/N 900,454, Affymetrix Santa Clara CA, USA) in Hybridization Oven 645 (P/N 00–0331 (220 V), Affymetrix Santa Clara CA, USA) and Fluidics Station 450 (P/N 00–0079, Affymetrix Santa Clara CA, USA) according to the manufacturer’s instructions. Arrays were scanned by GeneChip® Scanner 7G (Affymetrix, Santa Clara, CA, USA) using Command Console Software 3.2 (Affymetrix, Santa Clara, CA, USA) with default settings. Raw data was normalized by RMA and DABG algorithm, Expression Console (Affymetrix, Santa Clara, CA, USA).

### Rtranscription and quantitative real-time PCR

MiRNA and cDNA were synthesized from 500 ng total RNA by RevetAid RT Reverse Transcription Kit (Thermo Scientific, MA, USA) with specific miRNAs stem-loop RT primers (Table 1) and oligo d(T)_18_, using reverse transcription and quantitative real-time polymerase chain reaction (qRT-PCR). The miRNAs or mRNA expression levels were detected using Bio-Rad CFX96™ real-time PCR detection system (Bio-Rad, CA, USA) and each sample was detected in duplicate. Each assay consisted of 1 × SYBR Green quantitative real-time PCR master mix (Bio-Rad, CA, USA), 0.5 μM of forward and reverse primers (Table 1), and 1 μl cDNA template in a total volume of 20 μl. Non-template control was used as negative control, miRNAs reactions were normalized to *U6*, mRNA was normalized to *GAPDH* and the relative expression levels were calculated using the 2^−△Cq^ method.

**Table 1:** The list of primer sequences

Primer name	Forward primer (5′ → 3′)	Reverse primer (5′ → 3′)	Amplicon length (bp)	Tm (°C)
U6	CTCGCTTCGGCAGCACA	AACGCTTCACGAATTTGCGT	94	60
U6-RT	AACGCTTCACGAATTTGCGT			
miR-30a-5p	GCGCTGTAAACATCCTCGAC	GTGCAGGGTCCGAGGTATTC	60	60
miR-30a-5p-RT	GTCGTATCCAGTGCAGGGTCCGAGGTATTCGCACTGGATACGACCTTCCAGT			
miR-199-5p	GCCCAGTGTTCAGACTACCTG	GTGCAGGGTCCGAGGTATTC	58	60
miR-199-5p-RT	GTCGTATCCAGTGCAGGGTCCGAGGTATTCGCACTGGATACGACGAACAGGT			
miR-143-5p	GGTGCAGTGCTGCATCTCT	GTGCAGGGTCCGAGGTATTC	56	60
miR-143-5p-RT	GTCGTATCCAGTGCAGGGTCCGAGGTATTCGCACTGGATACGACACCA GAGA			
miR-486-3p	CGGGGCAGCTCAGTACAG	GTGCAGGGTCCGAGGTATTC	65	60
miR-486-3p-RT	GTCGTATCCAGTGCAGGGTCCGAGGTATTCGCACTGGATACGACATCCTGTA			
miR-199-3p	GCACAGTAGTCTGCACATTGG	GTGCAGGGTCCGAGGTATTC	68	60
miR-199-3p-RT	GTCGTATCCAGTGCAGGGTCCGAGGTATTCGCACTGGATACGACTAACCAAT			
miR-675-5p	TATATGGTGCGGAGAGGGC	GTGCAGGGTCCGAGGTATTC	71	60
miR-675-5p-RT	GTCGTATCCAGTGCAGGGTCCGAGGTATTCGCACTGGATACGACCACTGTGG			
DUSP5 mRNA	CTACTCGCTTGCCTACCC	ACATCCACGCAACACTCA	95	57
DUSP5 3′UTR	ATACTCGAGAACTGGGATGGAGGAATC	TGGGTCGACTGCTTTTCTCCTTTTACTATTTTTA	1118	53
MAP3K11 mRNA	GTGCCATACCGTGGCATTGA	CATTTCCCGTAGGACCTGTG	204	60
MAP3K11 3′UTR	GCTCGCTAGCCTCGAGGTGGGCCAGGCCACTCCC	ATGCCTGCAGGTCGACTGTGGGGAGACAGCTTTTGAG	536	67
CDH1 (E-cadherin)	GGATTGTCGGATTGGGAGAA	CATTCTGCTGCTTGAGGGTT	194	60
CDH2 (N-cadherin)	GAGTCGAACAGCAGCTCTGA	CTTGTCTGCTTCGGTTTGAC	199	60
VIM (Vimentin)	GATGTTTCCAAGCCTGACCT	CACTTCACAGGTGAGGGACT	216	60
COL1A1	GACTGGGAAACCAGATGCTG	GAAGCCTCTCTCTCCTCTCT	224	60
COL3A1	GAAGGAGGATGTTCCCATCT	GACCATTAGGAGGGCGAGTA	225	60
MMP1	GACTGGGAAACCAGATGCTG	GGTGAATGTCAGAGGTGTGA	226	60

### Cell culture

The human conjunctiva epithelial cell line (HConEpic, HCE) was purchased from BeNa Culture Collection (Beijing, China), which was primary culture cells (Additional file 1). HCEs were supplemented with High glucose Dulbecco’s modified Eagle’s medium (H-DMEM, HyClone) containing 10% fetal bovine serum (Gibco) and 1uM penicillin–streptomycin (Gibco). Cells were grown at a humidified atmosphere of 5% CO_2_ at 37 °C. After 24 h incubation in growth medium, TGF-β and EGF were added to obstain the optimal concentrations (10 nM & 20 nM). The medium was changed every other day and TGF-β and EGF were reintroduced to maintain the concentrations, and the cells that harvested after 7 days were used for subsequent experiments.

### Immunofluorescence staining

For EMT markers detection, HCEs were fixed with 3.5% paraformaldehyde, permeabilized with 0.1% Triton X-100, blocked with 2% bovine serum albumin (BSA, sigma, USA), and incubated over night at 4 °C with primary antibodies as following: anti-N-cadherin (1:100, Abclonal, China), anti-E-cadherin (1:100) and anti-vimentin (1:100). After washing with phosphate buffered saline (PBS, Gibco), the cells were incubated for 1 h with fluorescein isothiocyanate (FITC)-conjugated mouse immunoglobulin G secondary antibody (1:200). The stained cells were counterstained with 4′, 6-diamidino-2-phenylindole (DAPI, Invitrogen, USA) and viewed under a consistent fluorescence in situ hybridization (FISH) imager (BX51, Olympus, Tokyo, Japan).

### Plasmids construction and transient transfection

MiR-199a-3p and miR-199a-5p mimics and inhibitors were purchased from RiboBio Co. Ltd (Ribo, China) and short hairpin RNAs (shRNAs) knocking down *DUSP5* and *MAP3K11* were purchased from Shanghai Genechem Co. Ltd (Genechem, China) (Table 2). To overexpress *DUSP5* and *MAP3K11*, a full-length coding sequence (CDS) was amplified, and the EcoRI-HF and XhoI sites (NEB, USA) were used to insert the CDS product into pCMV-myc vector (Invitrogen, NY, USA). Before transfection, 2*10^5^ cells were seeded into each well of 6-well plates. After 24 h incubation in growth medium without penicillin–streptomycin, the cells were transiently transfected with miRNA mimics and inhibitors using riboFECT™ CP (Ribo, China), or with plasmids using Lipofectamine 2000 (Invitrogen, Carlsbad, CA, USA). The cells were incubated for an additional 24–48 h for the following experiments.

**Table 2:** The target sequence of DUSP5-shRNAs and MAP3K11-shRNAs

Name	Target sequence	Start site	GC (%)
DUSP5-shRNA1#	ctGAGTGTTGCGTGGATGTAA	620	47.37
DUSP5-shRNA2#	ccTGTCCTTCTGTGTGCTTAT	2213	42.11
DUSP5-shRNA3#	gaTAGGCCATTTGCAGACACT	1224	47.37
MAP3K11-shRNA1#	atTGAGAGTGACGACATGGAG	1250	52.63
MAP3K11-shRNA2#	tcTTCCCGTCCAACTATGTGT	774	47.37
MAP3K11-shRNA3#	ctTAGGAGGAGTCACAGCATA	3071	47.37

### Wound healing and transwell assay

HCEs dealt with TGF-β and EGF for 7d, were seeded equivalently into 6-well culture plates and then treated with transient transfection for 24 h before scratching or resuspending. A wound was scratched onto the monolayer with a sterile 20ul tip (Axygen, Union City, CA, USA). Images of HCEs migrating into the wound were captured at time points of 0, 24 and 48 h by an inverted microscope.

The migration assay was performed using upper chambers of Transwell insert (0.8um pore size, Corning Incorporated, Costar, USA) with 4*10^5^cells in serum-free medium for 48 h. After migration, cells passed through the coated membrane to the lower surface, where cell were fixed with 4% paraformaldehyde and stained with 0.1% crystal violet. The cell was counted under a microscope.

### Cell apoptosis determined by flow cytometry

The flow cytometry was performed to analyse the apoptosis of HCEs. Cells were re-suspended with Annexin V-FITC and propidiumiodide (PI) successively according to the manufacture’s protocol (BestBio, China) at the concentration of 10^6^ cells/ml. Cell apoptosis was analysed by flow cytometry (FACSCanto II; BD Bioscience, Franklin Lake, NJ).

### Western blot analysis

After cells were seeded in 6-well plates for 48–72 h, cells were washed with PBS and harvested in RIPA with phosphorylase inhibitor and phenylmethanesulfonyl fluoride (PMSF). Equal amounts of the supernatant were loaded per lane and resolved by SDS–polyacrylamide electrophoresis. Then, proteins were transferred onto polyvinylidene fluoride (PVDF) membrane (Millipore, USA) and blocked by 5% BSA. Primary antibodies should be probed overnight at 4 °C, including rabbit anti-MAP3K11, anti-DUSP5, anti-Vimentin, anti-E-Cadherin, anti-N-Cadherin antibodies and mouse anti-GAPDH (abClone) antibody. Membranes were washed in Tris buffered saline tween (TBS-T) and incubated with horseradish peroxidase-conjugated anti-rabbit or anti-mouse secondary antibodies. Membranes were washed in TBS-T and then exposed using the electrochemical luminescence (ECL) system. Protein loading was normalized by GAPDH.

### Dual-luciferase reporter assay

The DUSP5 or MAP3K11 fragments of 3′UTR whole region containing the wild-type binding sites of miR-199a -3p or miR-199a-5p, were cloned into pmirGLO vector (Promega, Madison, WI, USA) to generated DUSP5-WT and MAP3K11-WT vectors. Then the DUSP5-MUT, MAP3K11-MUT1, MAP3K11-MUT2 and MAP3K11-MUT1 + 2 vectors containing mutant loci were constructed, since there are 2 binding sites between miR-199a-5p and MAP3K11. For luciferase reporter assay, 293 T and HCE were plated in 12-well plates and transfected with miR-199a-3p or miR-199a-5p mimics and mimic-NC (50 nM) or miR-199a-3p or miR-199a-5p inhibitor and inhibitor-NC (100 nM), and 1ug of blank plasmids. After about 36 h of transfection, luciferase activity was detected by the Dual Luciferase Reporter Assay Kit (Promega, WI, USA) and the Promega GloMax 20/20 luminometer (Promega, WI, USA). All experiments were performed in triplicates and the relative luciferase activity ratios of firefly luciferase activity normalized to Renilla luciferase were calculated.

### MiRNAs target gene prediction and GO/pathway analysis

Differentially expressed miRNAs were subjected to target gene prediction analysis using TargetScan, miRDB, mirTarBase, miRanda, Targetminer and miRNAorg. The predicted results of target genes were shown by Venn diagram. GO network maps and term enrichment analysis were performed by using plug-in of Cytoscape: ClueGO and CluePedia, with terms defined by *GO_BiologicalProcess-GOA_07.12.2015 and KEGG pathway*. Significance was defined by *p* value < 0.05 and Kappa score threshold of 0.4 for pathways reporting.

### Statistical analysis

All data were analysed by GraphPad-Prism8.0 (Graph Pad, CA, USA) in independent t-test, Mann–Whitney U test or Pearson Correlation. *p* < 0.05 (two-tailed) was considered statistically significant.

## Result

### Expression profiling showed that miR-199a was upregulated in pterygium

**Fig. 1:**
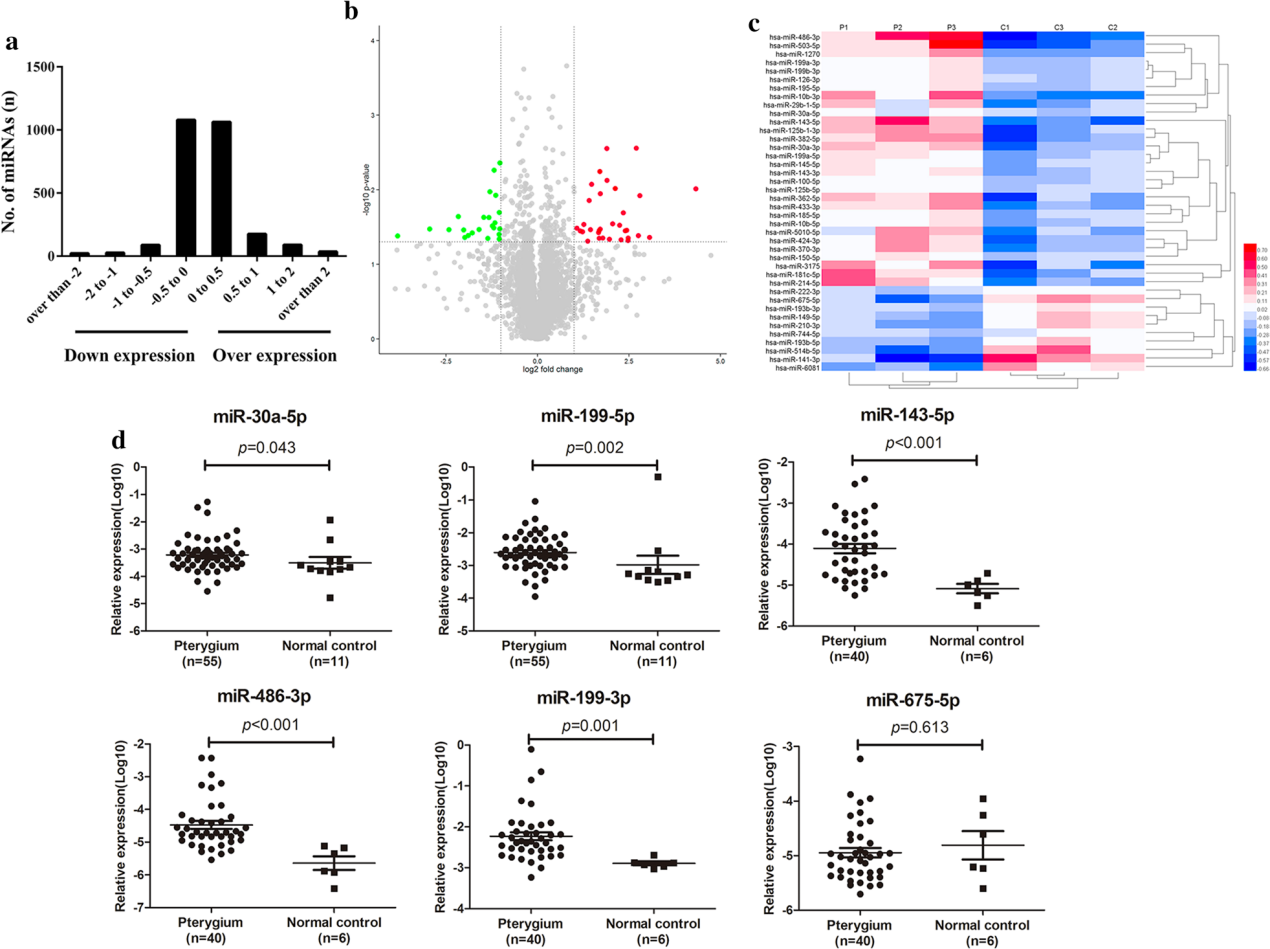
Differential expressed miRNAs in pterygium. **a** The log scales of most miRNAs expression in pterygium tissues were found from -1 to 1. X-axis indicates the log scale of miRNA expression in control conjunctiva and pterygium tissues. Negative scale indicates downregulation, and positive scale indicates overexpression. **b** The volcano plot of the microarray with the criteria of ∣lg (FC)∣ > 1 and *p* value < 0.05. The green nodes presented the downregulated miRNAs and the red nodes presented the upregulated miRNAs. **c** The heat map showed the expression levels of miRNAs which were indicated on a colour gradation scale from low expression (blue) to high expression (red) in pterygium (P1, P2, P3) and control conjunctiva (C1, C2, C3) groups. **d** The relative expression of six validated miRNAs in pterygium. MiR-30a-5p (*p* = 0.043) and miR-199a-5p (*p* = 0.002) were up-regulated in pterygium samples (n = 55) compared with control conjunctiva tissues (n = 11), and miR-143-5p (*p* < 0.0001), miR-486-3p (*p* < 0.0001), miR-199a-3p (*p* = 0.001) were up-regulated in pterygium samples (n = 40) compared with control conjunctiva tissues (n = 6). The verification result of miR-675-5p (*p* = 0.613) was inconsistent with the chip

To obtain miRNAs expression profiling in pterygium, miRNA microarray analysis was performed in 3 pairs of pterygium and control conjunctiva tissues. In the screened 2578 miRNAs, there were 1362 upregulated and 1216 downregulated miRNAs in pterygium, compared with control conjunctiva (Fig. 1a). Using *p* < 0.05 and ∣log_2_Fold Change (FC)∣ > 1 as the filtering criteria, there were 40 differentially expressed miRNAs in pterygium tissues were selected (Fig. 1b). These differentially expressed miRNAs were listed (Table 3) and analysed with heat map for visualization (Fig. 1c). Among them, 30 miRNAs were upregulated, and 10 miRNAs were downregulated.

**Table 3:** The list of 41 differentially expressed miRNAs

Gene symbol*	Fold change	p-values	Sequence length
hsa-miR-30a-5p	2.112	0.033	22
hsa-miR-30a-3p	4.872	0.047	22
hsa-miR-100-5p	4.741	0.030	22
hsa-miR-29b-1-5p	3.224	0.035	24
hsa-miR-199a-5p	3.910	0.046	23
hsa-miR-199a-3p	4.368	0.010	22
hsa-miR-10b-5p	3.733	0.007	23
hsa-miR-10b-3p	3.444	0.044	22
hsa-miR-181c-5p	3.283	0.034	22
hsa-miR-199b-3p	4.368	0.010	22
hsa-miR-214-5p	4.160	0.029	22
hsa-miR-125b-5p	2.725	0.034	22
hsa-miR-125b-1-3p	5.576	0.045	22
hsa-miR-143-5p	6.493	0.003	22
hsa-miR-143-3p	6.958	0.012	21
hsa-miR-145-5p	5.561	0.048	23
hsa-miR-126-3p	3.288	0.011	22
hsa-miR-150-5p	2.789	0.008	22
hsa-miR-185-5p	5.271	0.036	22
hsa-miR-195-5p	3.714	0.003	21
hsa-miR-362-5p	5.418	0.035	24
hsa-miR-370-3p	2.406	0.029	22
hsa-miR-382-5p	6.757	0.041	22
hsa-miR-424-3p	3.182	0.037	21
hsa-miR-433-3p	2.661	0.014	22
hsa-miR-486-3p	20.11	0.010	21
hsa-miR-503-5p	8.349	0.044	23
hsa-miR-1270	2.247	0.036	23
hsa-miR-3175	5.079	0.020	22
hsa-miR-5010-5p	3.225	0.045	22
hsa-miR-210-3p	0.247	0.035	22
hsa-miR-222-3p	0.391	0.045	21
hsa-miR-141-3p	0.071	0.042	22
hsa-miR-149-5p	0.223	0.023	23
hsa-miR-193b-5p	0.270	0.041	22
hsa-miR-193b-3p	0.331	0.034	22
hsa-miR-675-5p	0.130	0.034	23
hsa-miR-744-5p	0.446	0.028	22
hsa-miR-514b-5p	0.187	0.034	22
hsa-miR-6081	0.486	0.020	24

^*^Using filtering criteria of p < 0.05 and two fold change, 40 miRNAs were differentially expressed

Furthermore, some of the differentially expressed miRNAs were verified in a small group of samples as the explorer category, including 55 pterygium samples and 12 control conjunctiva tissues. Coincident with the results of miRNA microarray, the qRT-PCR verified the differential expressed miRNAs except for miR-675-5p, which presented no significant decrease in the 55 pterygium samples (*p* > 0.05) (Fig. 1d). Based on the essential role of miR-199 family in the EMT process, and the primary exploration results of microarray, miR-199a-3p and miR-199a-5p were selected for further exploration.

### The EMT cell model was established using HCEs induced by TGF-β and EGF

**Fig. 2:**
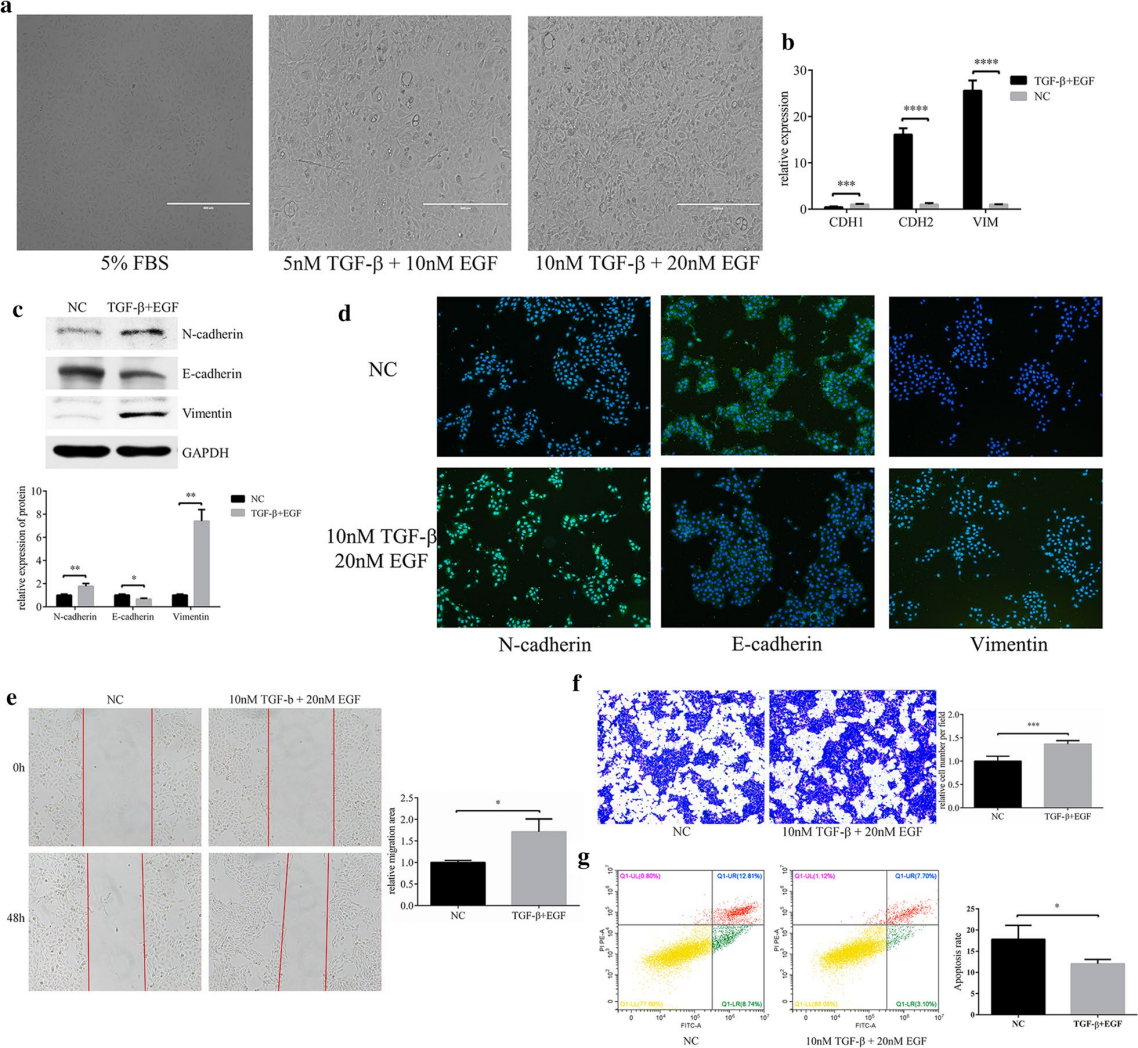
TGF-β and EGF could induced EMT in HCEs. **a** A combination of TGF-β 10 nM and EGF (20 nM) could induced HCEs to show an EMT phenotype. **b**–**d** The changes in EMT markers were detected with qRT-PCR, western blot and immunofluorescence (*p < 0.05, **p < 0.01, ***p < 0.001, ****p < 0.0001). **e**, **f** Cell migration in HCEs induced by TGF-β (10 nM) and EGF (20 nM). Cell migration was determined by the wound healing assay and transwell assay (*p < 0.05, ***p < 0.001). **g** Apoptosis in HCEs induced by TGF-β (10 nM) and EGF (20 nM). The apoptosis cells were decreased compared with the HCEs without inducement (*p < 0.05)

We attempted to establish the pterygium EMT-cell model in HCEs, by using TGF-β and EGF stimulation. After cultured in the inducement of 10 nM TGF-β and 20 nM EGF for 7 days, the HCEs grew in a long spindle shape, showing a mesenchymal cell phenotype (Fig. 2a). The characteristic of EMT-HCEs was identified by EMT markers detected by qRT-PCR, western blot and immunofluorescence. Compared with control HCEs, the expression level of N-cadherin and Vimentin increased significantly, while E-cadherin decreased (Fig. 2b–d). Then, the wound healing and transwell assays were executed and found that the migration ability of induced cells was also improved after EMT activation (Fig. 2e, f). Furthermore, decreased cell apoptosis was observed by flow cytometry with the Annexin V-FITC/PI reagent (Fig. 2g).

To further investigate that whether the EMT-HCEs model could represent the EMT process in pterygium, we compared expressions of EMT markers between primary culture pterygium cells and HCEs and found the EMT markers were significantly lower in the latter (Additional file 2).

### Overexpression of miR-199a-3p/5p promoted EMT in HCEs

**Fig. 3:**
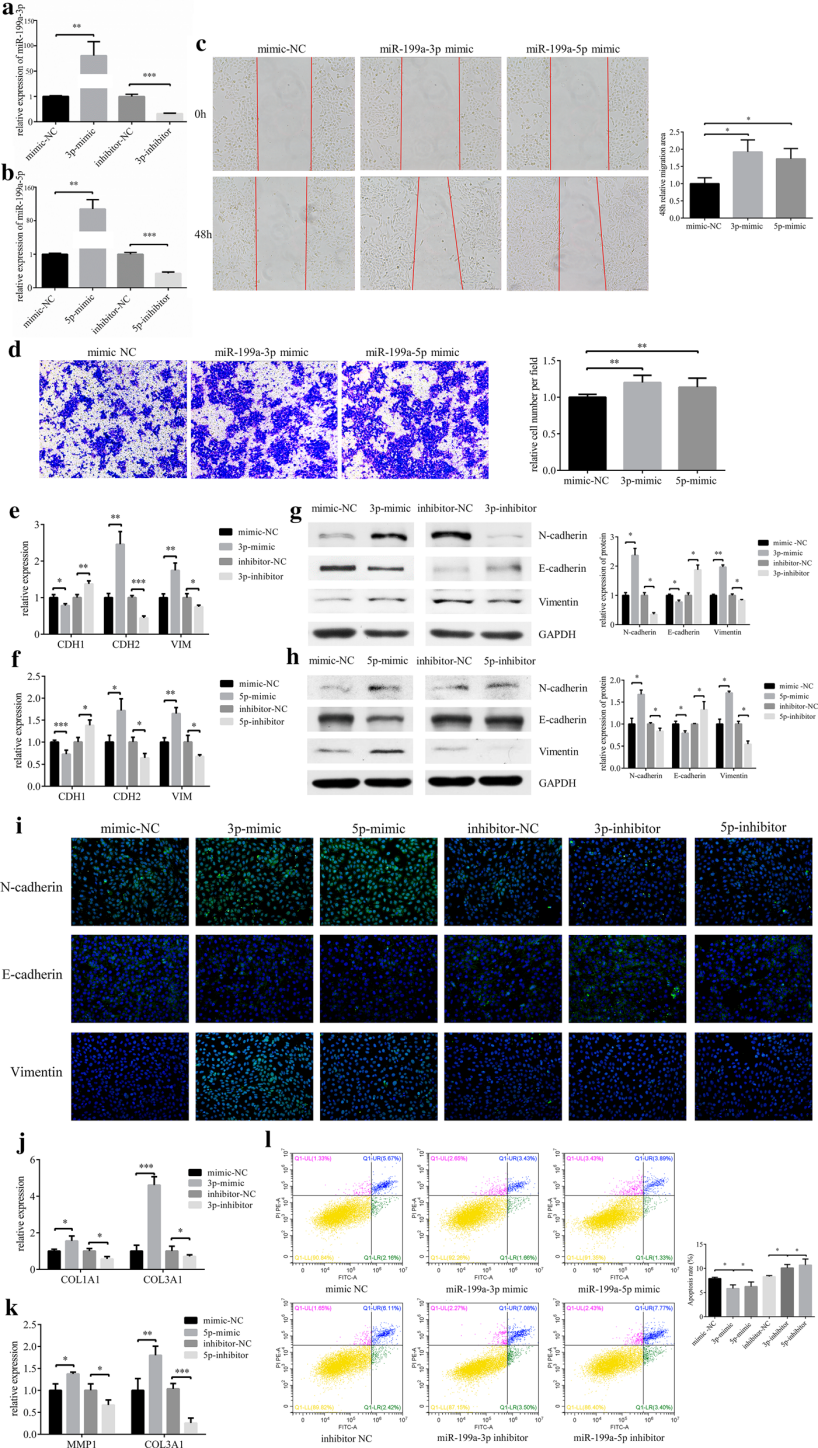
MiR-199a-3p and miR-199a-5p promotes migration of EMT in HCEs. **a**, **b** HCEs were transfected with miR-199a-3p/miR-199a-5p mimics/mimics NC, inhibitors/inhibitor NC. The expression of miR-199a-3p and miR-199a-5p was detected by qRT-PCR (**p < 0.01, ***p < 0.001). HCEs were transfected with miR-199a-3p mimic, miR-199a-5p mimic and mimic NC. **c** Cell migration was determined by the wound healing assay (*p < 0.05). **d** Cell migration was determined by the transwell assay (**p < 0.01). **e**–**i** HCEs were transfected with miR-199a-3p/miR-199a-5p mimic, mimic NC, miR-199a-3p/miR-199a-5p inhibitor and inhibitor NC. The EMT markers (E-cadherin (CDH1), N-cadherin (CDH2) and Vimentin (VIM)) were detected by qRT-PCR, western blot and immunofluorescence (*p < 0.05, **p < 0.01, ***p < 0.001). **j** After HCEs were transfected with miR-199a-3p mimic, mimic NC, miR-199a-3p inhibitor and inhibitor NC, COL1A1 and COL3A1 were upregulated only by miR-199a-3p, detected by qRT-PCR (*p < 0.05, ***p < 0.001). **k** After HCEs were transfected with miR-199a-5p mimic, mimic NC, miR-199a-5p inhibitor and inhibitor NC, MMP1 and COL3A1 were upregulated only by miR-199a-5p, detected by qRT-PCR (*p < 0.05, **p < 0.01, ***p < 0.001). **l** HCEs were transfected with miR-199a-3p/miR-199a-5p mimics/mimics NC, inhibitors/inhibitor NC. Transfection of the miR-199a-3p mimic and miR-199a-5p mimic decreased the apoptosis cells, while miR-199a-3p inhibitor and miR-199a-5p inhibitor increased the apoptosis cells (*p < 0.05, **p < 0.01)

We used commercially obtained mimics for miR-199a-3p and miR-199a-5p overexpression, and inhibitors for knockdown (Fig. 3a and b). Transfected with miR-199a-3p or miR-199a-5p mimics respectively, resulted in a significant increase in cell migration ability (Fig. 3c and d) and promoted the occurrence of EMT (Fig. 3e–h). Immunofluorescence was used to verify the result of western blot (Fig. 3i). N-cadherin and Vimentin expressions were increased, while E-cadherin was reduced, when HCEs treated with miR-199a-3p/5p mimics. Then we detected some matrix metalloproteinases and collagens to explore the extracellular matrix environment. We found both miR-199a-3p and miR-199a-5p upregulated the expression of COL3A1 (Fig. 3j and k). Overall, the results showed that both miR-199a-3p and miR-199a-5p promoted the incidence of EMT in HCEs. Transfected with miR-199a-3p/5p mimics resulted in a significant suppression in cell apoptosis (Fig. 3l).

### Knockdown miR-199a-3p/5p hindered EMT

**Fig. 4:**
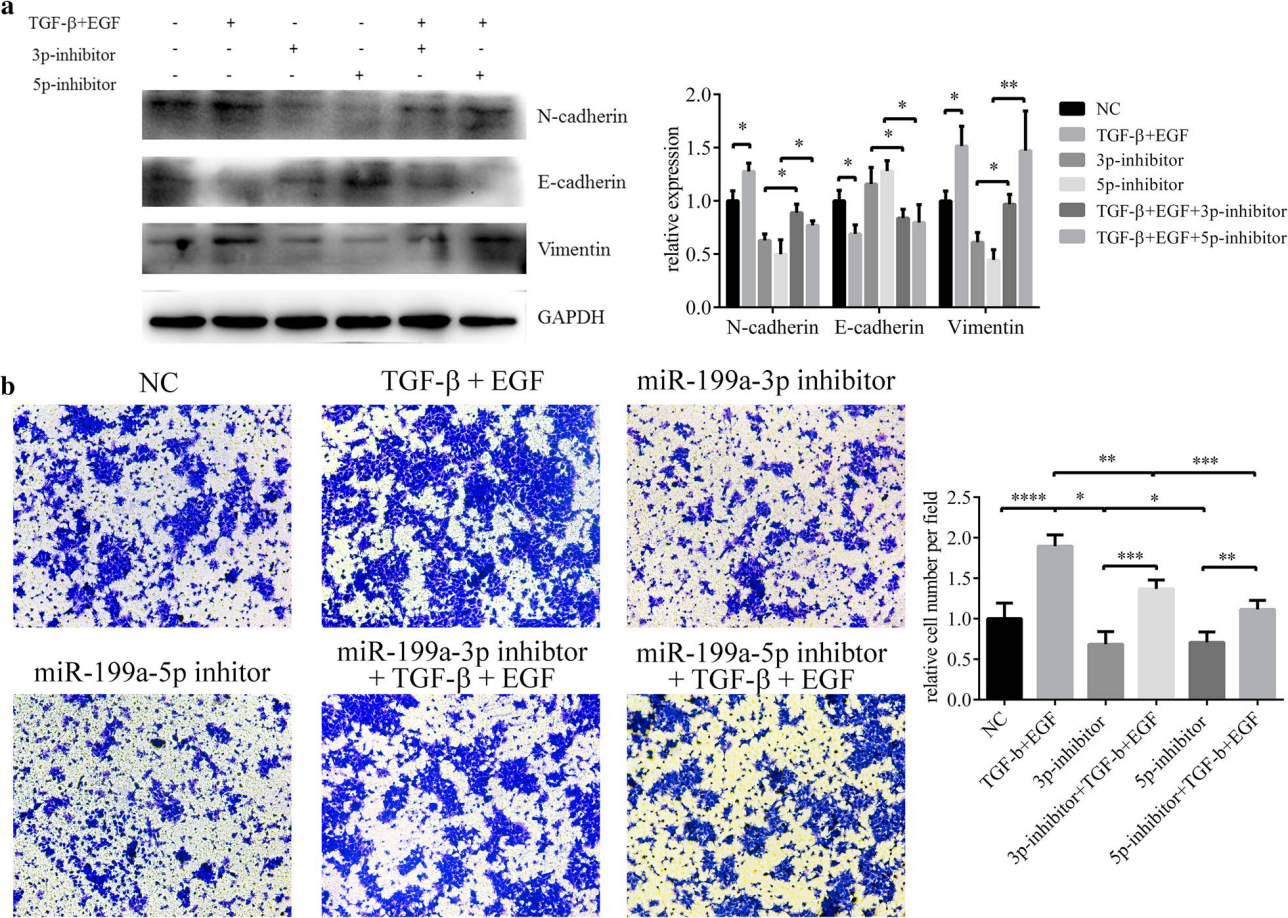
MiR-199a inhibitor hindered TGF-β and EGF induced EMT in HCEs. **a** The changes of EMT biomarkers detected with western blot (*p < 0.05, **p < 0.01). **b** The migration ability of HCEs measured by transwell assay (*p < 0.05, **p < 0.01, ***p < 0.001, ****p < 0.0001)

TGF-β & EGF and miR-199a-3p/5p both induced EMT in HCEs. But after transfection with miR-199a-3p and miR-199a-5p inhibitor, features of EMT in induced HCEs were diminished (Fig. 4a), so did the migration ability (Fig. 4b). Our result further suggested miR-199a-3p or miR-199a-5p promoted the EMT process of HCEs (Additional file 3).

### DUSP5 and MAP3K11 were the targets of miR-199a-3p and miR-199a-5p

**Fig. 5:**
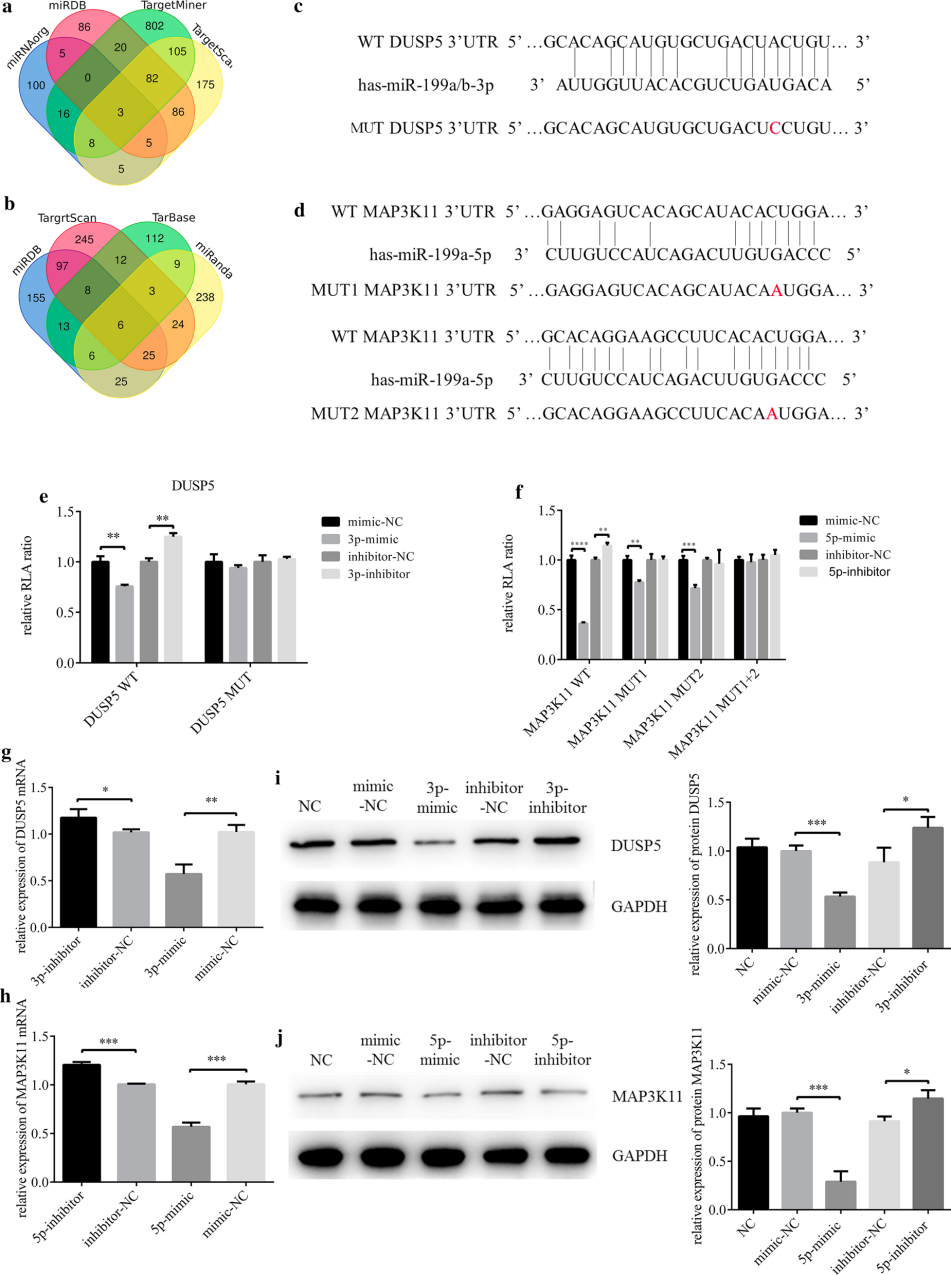
MiR-199a-3p and miR-199a-5p target DUSP5 and MAP3K11 respectively. **a**, **b** Venn diagram of miR-199a-3p and miR-199a-5p target genes prediction results from 4 databases. **c**, **d** Putative miR-199a-3p and miR-199a-5p binding sites in the 3′UTR of DUSP5 and MAP3K11, and the designed mutation sites. **e**, **f** A luciferase reporter plasmid containing wild type or mutant type were co-transfected with relevant mimic, mimic NC, inhibitor and inhibitor NC into HCEs. Luciferase activity was normalized to Renilla luciferase activity. Of note, MAP3K11 3′UTR had 2 binding sites withmiR-199a-5p, so 3 kinds of mutants were construct, including mutant at the first site, mutant at the second site and mutant at both sites (**p < 0.01, ***p < 0.001, ****p < 0.0001). **g**–**j** After HCEs were transfected with miR-199a-3p/miR-199a-5p mimic, mimic NC, miR-199a-3p/miR-199a-5p inhibitor and inhibitor NC, the variations of DUSP5 and MAP3K11 expression were analyzed with qRT-PCR and western blot (*p < 0.05, **p < 0.01, ***p < 0.001)

The predicted target genes DUSP5 and MAP3K11 were first selected from multiple miRNA related databases, then filtrated with Venn diagram (Fig. 5a and b). The predicted binding between the 3′-untranslated region (3′-UTR) of DUSP5/MAP3K11 and miR-199a-3p/5p were confirmed by dual-luciferase reporter gene assay (Fig. 5c and d). Luciferase activity of DUSP5-WT 3′UTR could be inhibited by miR-199a-3p mimic, but DUSP5-MUT 3′UTR could not (Fig. 5e). There were 2 predicted binding sites between the 3′UTR of MAP3K11 and miR-199a-5p, and luciferase activity of MAP3K11-WT (wild type) 3′UTR was inhibited by miR-199a-5p mimic. With mutation at either of the two binding sites (MAP3K11-MUT1 and MAP3K11-MUT2), luciferase activity could still be inhibited by mimic, but the inhibition rate was approximately 50% while with mutations at both binding sites (MAP3K11-MUT1 + 2) it was nearly 100% (Fig. 5f). The negative correlation between miR-199a-3p/5p and their target genes in both mRNA and protein levels were observed in tissues and cell models to further verify that miR-199a-3p/5p downregulated the expression of DUSP5 and MAP3K11 (Fig. 5g–j).

### DUSP5/MAP3K11 inhibition could promote EMT in HCEs

**Fig. 6:**
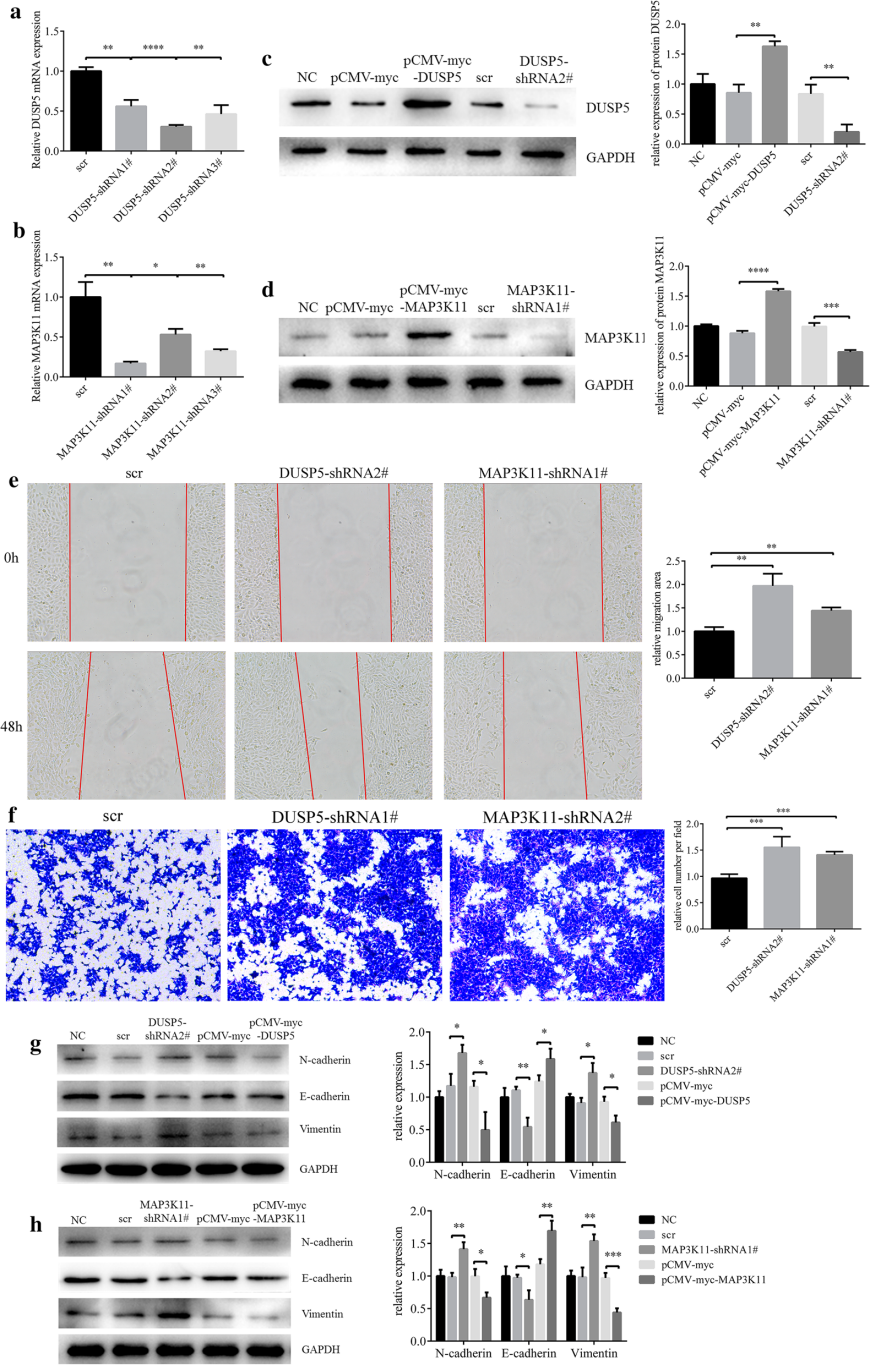
Downregulating DUSP5 or MAP3K11could promote HCEs migration and EMT. **a, b** The shRNAs which had the best knockdown efficiency were selected with qRT-PCR. **c, d** The effect of pCMV-myc-DUSP5, pCMV-myc-MAP3K11, DUSP5-shRNA2# and MAP3K11-shRNA#1. HCEs were transfected with pCMV-myc and scr as control. **e** Cell migration was determined by the wound healing assay (**p < 0.01). **f** Cell migration was determined by the transwell assay (**p < 0.01). **g**, **h** The changes in EMT markers, after HCEs were transfected with pCMV-myc-DUSP5, pCMV-myc-MAP3K11, pCMV-myc, DUSP5-shRNA2#, MAP3K11-shRNA#1 and shRNA control. (*p < 0.05, **p < 0.01, ***p < 0.001)

It was shown that DUSP5-shRNA2# and MAP3K11-shRNA1# had the best effects on DUSP5/MAP3K11 knockdown, and both of the overexpression vectors demonstrate significantly upregulations of the two gene (Fig. 6a–d). Some HCEs were treated with TGF-β and EGF for 36 h to extract RNA, while the others were treated for 48 h to extract protein or use for wound healing and transwell assay. (Fig. 6e and f). Knockdown of either DUSP5 or MAP3K11 promoted the HCEs migration, and when either of the two genes was overexpressed cell motility was inhibited (Fig. 6g and h).

### Suppression on DUSP5/MAP3K11 induced by TGF-β and EGF could be alleviated by decreased miR-199a-3p/5p expression

**Fig. 7:**
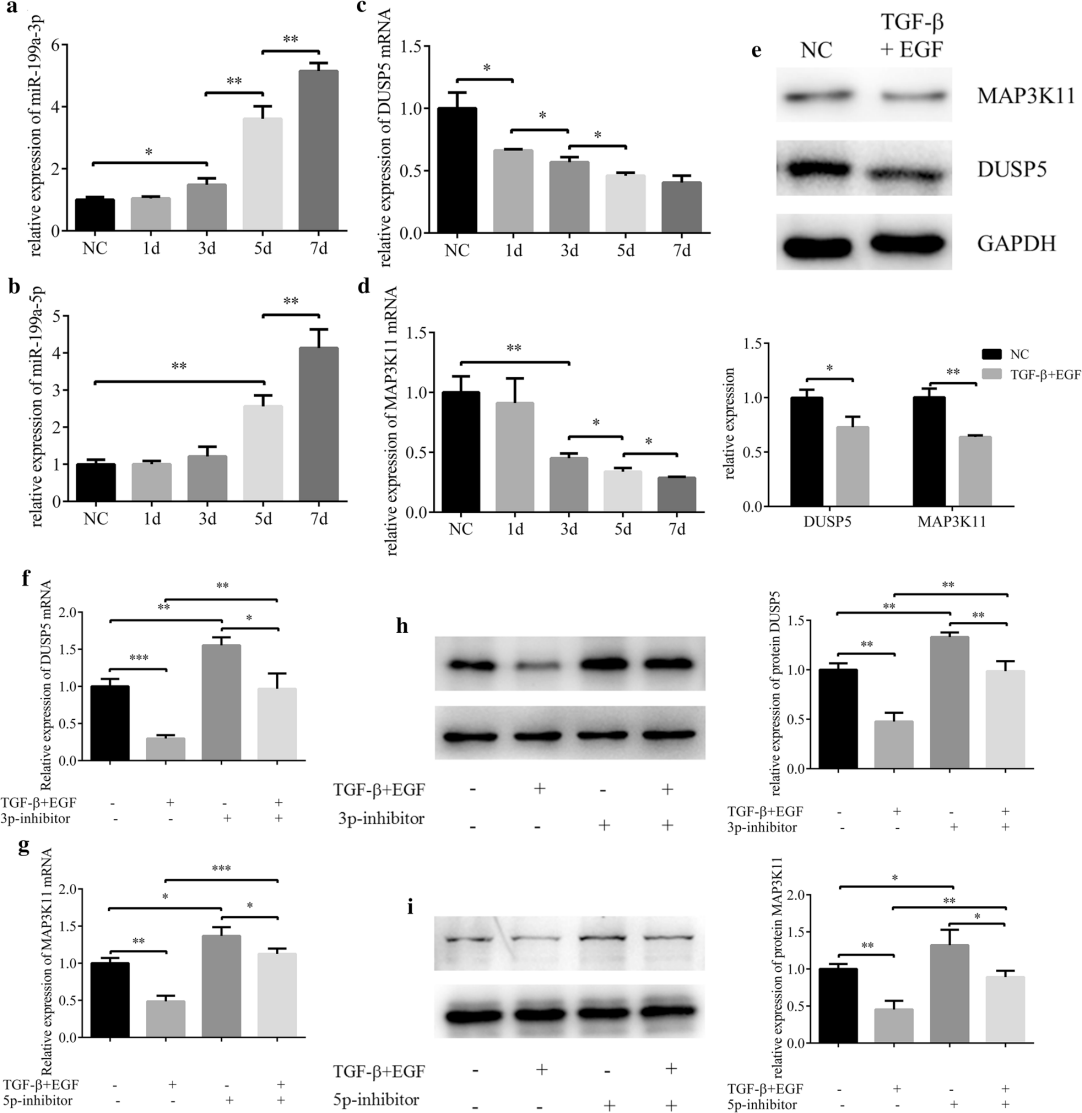
The effects of TGF-β and EGF on expressions of DUSP5 and MAP3K11 could be regulated by miR-199a-3p and miR-199a-5p. **a**–**d** HCEs were induced with TGF-β (10 nM) and EGF (20 nM) for 7 days. The expressions of miR-199a-3p, miR-199a-5p, DUSP5 and MAP3K11 were detected by qRT-PCR analysis (*p < 0.05, **p < 0.01). **e** The protein expression of DUSP5 and MAP3K11 were analyzed with western blot (*p < 0.05, **p < 0.01). **f**, **g**. The mRNA changes of DUSP5 and MAP3K11 before and after induction and transfection (*p < 0.05, **p < 0.01, ***p < 0.001). **h**, **i** The protein changes of DUSP5 and MAP3K11 before and after induction and transfection (*p < 0.05, **p < 0.01)

Along with the prolongation of TGF-β and EGF induction, the expressions of miR-199a-3p and miR-199a-5p were increased, while the expressions of DUSP5 and MAP3K11 were decreased. Moreover, compared with HCE, the expression levels of DUSP5 and MAP3K11 of induced HCEs were significantly reduced (Fig. 7a–e). However, inhibiting the expression of miR-199a-3p/5p in EMT-HCEs resulted in increased expression of corresponding target genes (Fig. 7f–i). That is to say, the inhibition on DUSP5 and MAP3K11 expressions induced by TGF-β and EGF was weakened by decreasing the expression of miR-199a-3p or miR-199a-5p. So, we speculated that miR-199a-3p and miR-199a-5p might target DUSP5 and MAP3K11 to function in EMT of pterygium.

### MiR-199a-3p/5p potentiated migration in EMT of HCEs induced by TGF-β and EGF by targeting DUSP5/MAP3K11

**Fig. 8:**
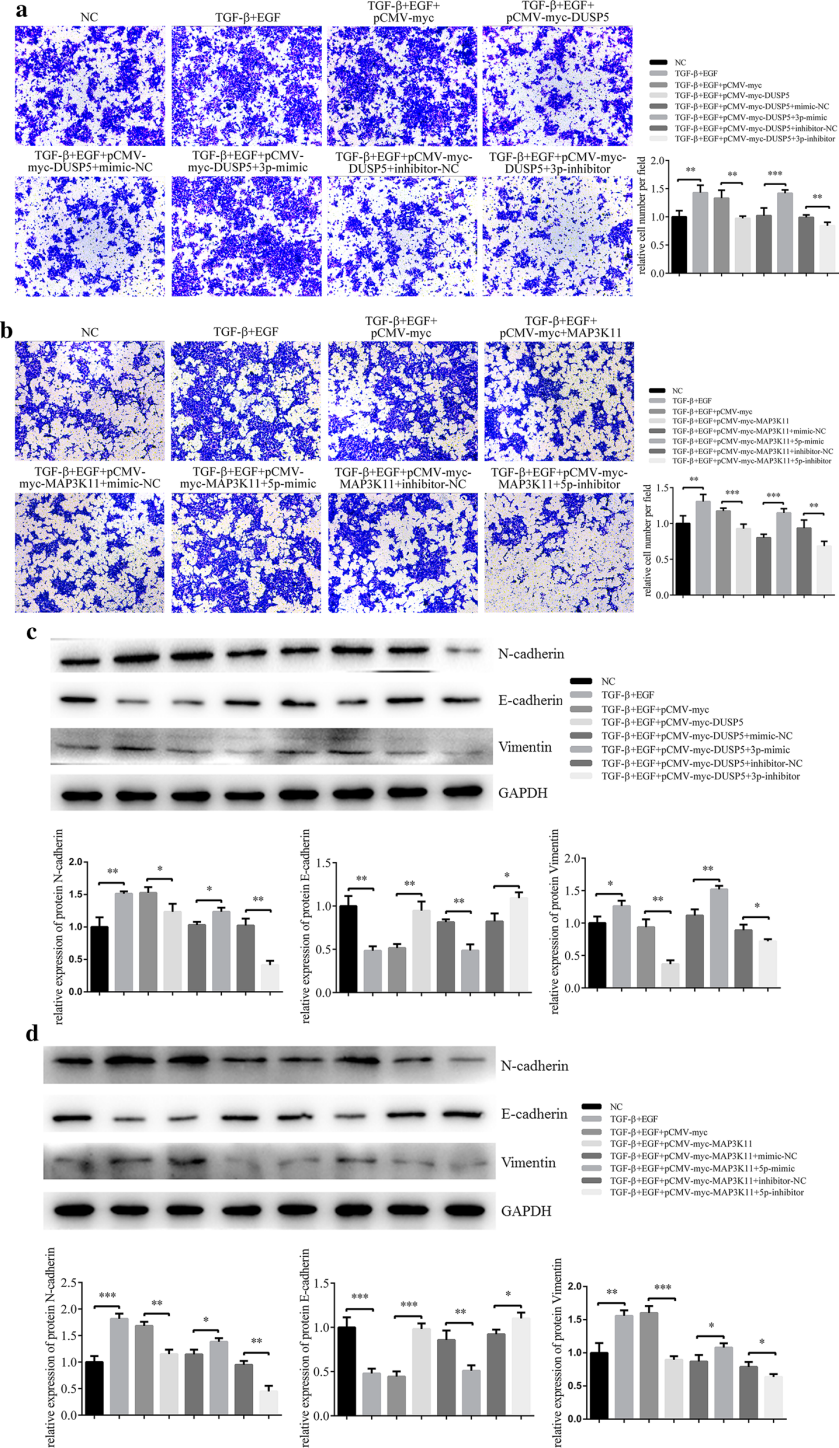
Inhibition on miR-199a-3p and miR-199a-5p alleviates the EMT promoted by DUSP5 and MAP3K11 in induced HCEs. HCEs were induced with TGF-β (10 nM) and EGF (20 nM) for 7 days, and subsequently transfected with pCMV-myc-DUSP5 (or pCMV-myc-MAP3K11) and pCMV-myc, miR-199a-3p (miR-199a-5p) inhibitor/inhibitor NC and miR-199a-3p (miR-199a-5p) mimic/mimic NC. Un-transfected HCEs with or without inducement were also included. **a, b** The migration ability of HCE was measured by transwell assay (**p < 0.01, ***p < 0.001). **c, d** The EMT progression of HCE was measured as 3 EMT markers by western blot (*p < 0.05, **p < 0.01, ***p < 0.001)

Transwell assays were then used to determine the effect of miR-199a-3p/5p-DUSP5/MAP3K11 axes regulation on TGF-β and EGF-induced migration of HCEs. Treated with 10 nM TGF-β and 20 nM EGF for 7 days, the migration ability of HCEs was increased, while the effects were inhibited when either of DUSP5 or MAP3K11 was overexpressed. The migration ability of HCEs was enhanced when miR-199a-3p or miR-199a-5p was overexpressed by mimics but decreased when either of the two miRNAs was knockdown by inhibitors (Fig. 8a and b). The inhibition of miR-199a-3p or miR-199a-5p resulted in the enhancement of DUSP5 or MAP3K11-mediated inhibition on EMT-HCEs migration, while conversely promoting miR-199a-3p or miR-199a-5p could further induce migration of EMT-HCEs. The change trend of EMT markers was consistent with that of migration ability. Overexpression of DUSP5 or MAP3K11 rescued the effects of TGF-β and EGF-induced EMT, while miR-199a-3p/5p could further promote the induction by regulating the expression of DUSP5/MAP3K11 respectively (Fig. 8c and d). In short, the promotion of EMT by miR-199a-3p/5p was achieved by regulating target genes DUSP5/MAP3K11.

### MiR-199a-3p-DUSP5 and miR-199a-5p-MAP3K11 axes regulated EMT in pterygium

**Fig. 9:**
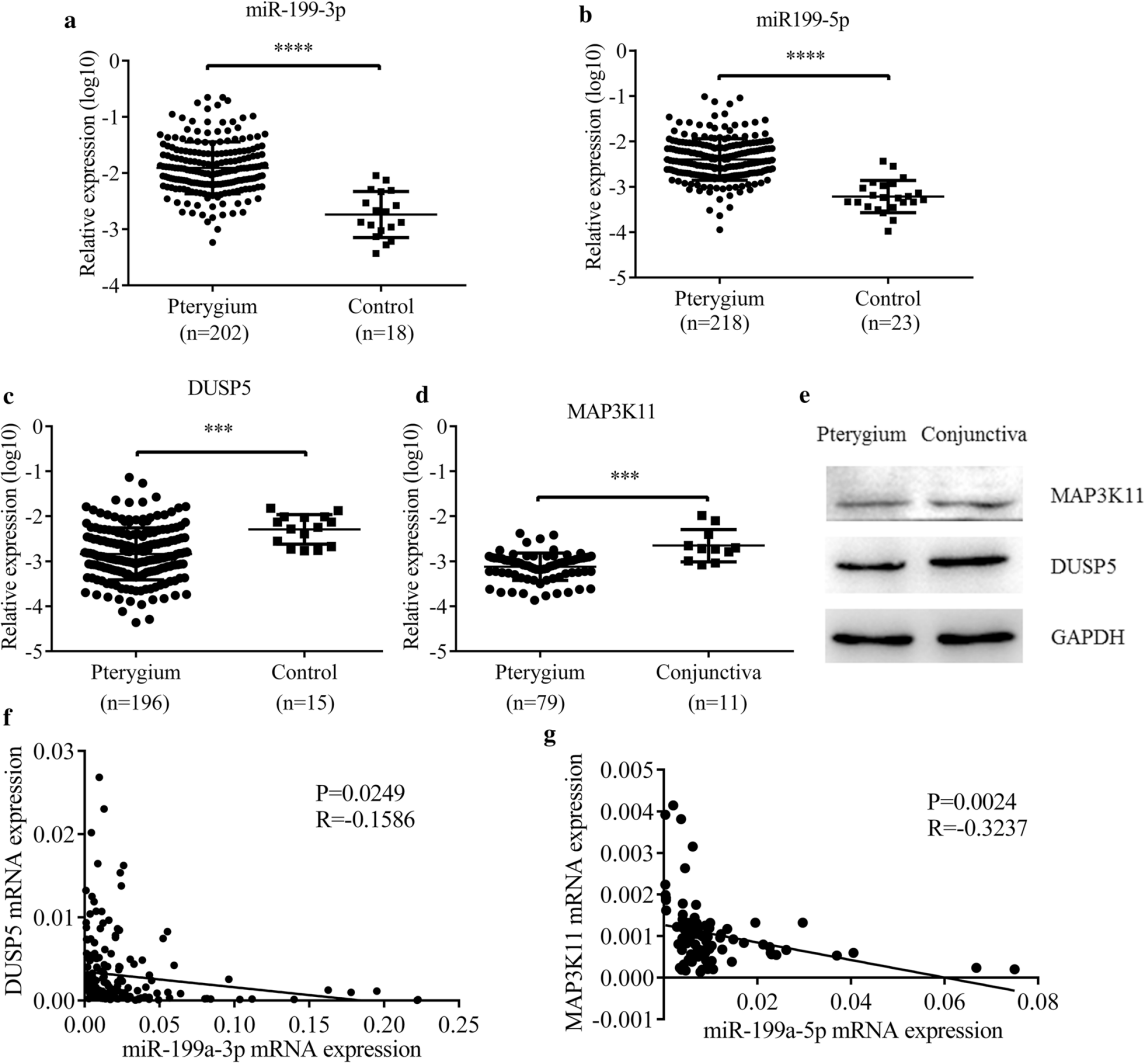
MiR-199a-3p, miR-199a-5p, DUSP5 and MAP3K11 expressions in pterygium. **a**, **b** Both miR-199a-3p (****p < 0.0001) and miR-199a-5p (****p < 0.0001) were upregulated in pterygium samples (n = 234) compared with control conjunctiva tissues (n = 29). **c**, **d** The relative expression of *DUSP5* mRNA was downregulated in pterygium samples (n = 196) compared with control conjunctiva tissues (n = 15) (***p < 0.001). The relative expression of *MAP3K11* mRNA was downregulated in pterygium samples (n = 79) compared with control conjunctiva tissues (n = 11) (***p < 0.001). **e** DUSP5 and MAP3K11 expressions were analyzed by western blot in pterygium samples and control conjunctiva tissues. **f**, **g** The relationship between *DUSP5* mRNA and miR-199a-3p expressions, *MAP3K11* mRNA and miR-199a-5p expressions were detected by Spearman’s correlation analysis, and both of them significantly showed a negative correlation (R = -0.1586, *P* = 0.0249; R = -0.3237, *p* = 0.0024)

In order to further confirm the function of miR-199a-3p and miR-199a-5p in pterygium, qRT-PCR was performed in a large number of pterygium samples (n = 234) and control conjunctiva tissues (n = 29). Both expressions of miR-199a-3p and miR-199a-5p were significantly increased in pterygium (p < 0.0001) (Fig. 9a and b), while expressions of DUSP5 (p = 0.0124) and MAP3K11 (p = 0.0005) were significantly decreased in pterygium (Fig. 9c and d). Downregulated protein expressions of DUSP5 and MAP3K11 in pterygium tissues were verified by western blot (Fig. 9e). Moreover, expressions of miR-199a-3p and miR-199a-5p tended to be negatively associated with those of DUSP5 and MAP3K11 respectively (Fig. 9f and 9g), which is coincident with the previous experiments in HCEs.

### Downstream pathway prediction by bioinformatics

**Fig. 10:**
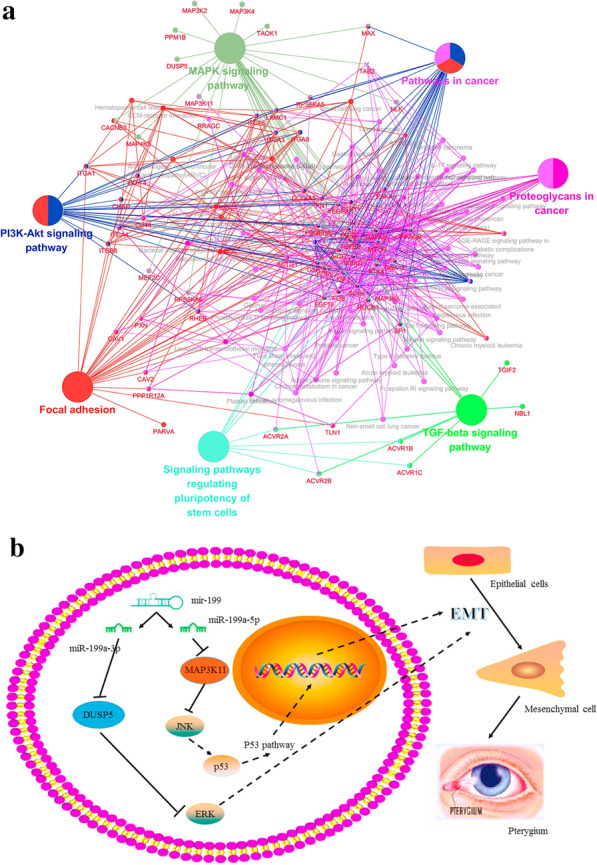
MiR-199a-3p and miR-199a-5p might affect MAPK signaling pathway by regulating DUSP5 and MAP3K11. **a** KEGG pathway network for miR-199a-3p and miR-199a-5p. Genes that enrich for selected terms are displayed as small nodes connecting to the larger pathway nodes. Both of DUSP5 and MAP3K11 were related to MAPK signaling pathway. **b** Based on KEGG pathway analysis, miR-199a-3p and miR-199a-5p had an effect on EMT process through MAPK by targeting DUSP5 or MAP3K11

In order to explore the possible downstream pathway of miR-199a-3p and miR-199a-5p in greater depth in the occurrence and development of EMT in pterygium, we subjected the potential targets of the two miRNAs to pathway enrichment analysis. The network showed that miR-199a-3p and miR-199a-5p might participate in the development of pterygium by affecting MAPK signalling pathway, TGF-β signalling pathway, PI3K-Akt signalling pathway, focal adhesion and others (Fig. 10a). From the pathway enrichment network, both of the DUSP5 and MAP3K11 were related with MAPK signalling pathway and we speculated that miR-199a might be involved in the MAPK pathway by targeting DUSP5 and MAP3K11, to promote EMT in HCE. Consistent with other researches, both of miR-199a-3p and miR-199a-5p had the positive effect on EMT processes and induced cell migration through MAPK signalling pathway, such as miR-199a-3p-DUSP5-ERK and miR-199a-5p-MAP3K11-JNK-p53 pathway. The pathway network was established based on our wet-lab experiments and dry-lab analysis (Fig. 10b).

## Discussion

MiR-199a-3p-DUSP5 and miR-199a-5p-MAP3K11 axes regulated EMT in pterygium were characterized in our study. In accordance with previous researches, both activated TGF-β and EGF stimulated the process of migration, proliferation and EMT in many different diseases [36–38], our study proved the feasibility of building EMT cell model using TGF-β and EGF. Furthermore, it presented that miR-199a-3p and miR-199a-5p participated in the process of EMT induced by TGF-β and EGF, by targeting DUSP5 and MAP3K11 in pterygium.

First, we got the miRNA expression profile of pterygium using the Affymetrix’ miRNA 4.0 microarray and validated it in pterygium samples, where we first observed the significant increase of miR-199a in pterygium. Although there were several researches, using microarray to screen the differentially expressed miRNAs in pterygium [2, 26–28], the Affymetrix’ miRNA 4.0 microarray provided more complete measurements of miRNA, compared to GeneChip® miRNA 2.0 and 3.0, based on reports in Sanger miRBase 20.0 database. For instance, a larger number of miRNAs reads, reached to 6659, were discovered in our study compared to previous pterygium microarray researches, such as Silin Chen’s (GSE21346), only 1380 reads.

MiR-199 family, consisting of miR-199a/b-3p and miR-199a/b-5p, is encoded within the Dynamin (DNM) genes and exhibit high conservation across species [39]. MiR-199a-3p/5p are found to inhibit EMT in various cancers, while they also can activate EMT in fibrosis [15, 34, 35]. The reason that why miR-199a-3p/5p have different expression level in EMT lies in the intrinsic mechanism of different types of EMT [12].

Second, miR-199 family functioning in pterygium is consist of miR-199a/b-3p and miR-199a/b-5p, and encoded within the Dynamin (DNM) genes, which exhibits high conservation across species [39]. MiR-199a-3p/5p are found to inhibit EMT in various cancers, while they also can activate EMT in fibrosis diseases [15, 34, 35]. The reason that why miR-199a-3p/5p have contradiction functions in EMT of pterygium compared with cancers, probably due to the essential fibrosis characteristic of pterygium [12].

Third, there are mounting evidences suggesting that miR-199 family acts as a fatal effector of TGF-β signalling in many diseases, regulating multiple disordered processes, including cell proliferation, apoptosis, migration, invasion and EMT [15, 34, 40]. The most typical biomarker change in EMT was the conversation from E-cadherin to N-cadherin, accompanied by the increase of cell migration ability and the decrease of cell apoptosis [41]. We found that as increasing time of TGF-β and EGF treatment, expressions of both miR-199a-3p and miR-199a-5p were increased gradually. The EMT characteristics including migration etc. of induced HCEs could be suppressed by inhibiting the expression of either miR-199a-3p or miR-199a-5p. It disclosed that miR-199a-3p/5p possibly acted as TGF-β effector in pterygium.

Forth, Antoon JW et al. recently found that inhibition of p38 mitogen-activated protein kinase (MAPK) signalling pathway even could reverse EMT [42], which is previously considered as a key process of inducting and maintaining the inflammation in various disease [43]. Accordingly, our study found that both *DUSP5* and *MAP3K11* implicated in MAPK signalling pathway, might be a potential therapeutic agent targeted specifically to reverse EMT in pterygium. Consistently, knockdown DUSP5 or MAP3K11 promoted EMT in the present study. However, upregulated DUSP5 inhibited the process of EMT in gastric cancer and hepatocellular carcinoma through MAPK pathway, and the cells showed a reduced migration ability and increased apoptosis [44, 45]. Furthermore, combining with existing literature, our pathway bioinformatics analysis also speculated that *DUSP5* and *MAP3K11* function as a suppressor in cell EMT by downregulating ERK and JNK [32, 46, 47].

Fifth, with increasing induction time of TGF-β and EGF, expressions of DUSP5 and MAP3K11 were decreased in accordance with the increase of miR-199a. The results of compensation showed that, the promotion of miR-199a on EMT was possibly executed through targeting DUSP5 and MAP3K11. Regardless effects of miR-199a-3p and miR-199a-5p, high expressions of DUSP5 or MAP3K11 could inhibit the EMT. That is to say, TGF-β and EGF affected the expression of DUSP5 and MAP3K11 through regulations on expressions of miR-199a-3p and miR-199a-5p, so as to take part in the MAPK signalling pathway, further to promote the EMT of HCEs, and participate in the initiation and development of pterygium.

However, there were still some limitations in the present study. Firstly, the number of control conjunctiva tissues was limited. Secondly, the investigation on large population in different stages of pterygium might be helpful for understanding miRNA’s function in different stages of pterygium [6, 25]. Finally, the in vivo animal experiment was lack.

## Conclusions

Our present research presented that TGF-β and EGF activated the miR-199a-3p/5p-DUSP5/MAP3K11-MAPK axes in the EMT process of pterygium. Our research results supplemented basic understanding of the pathophysiological mechanisms of pterygium, hoping to help develop new targets for pterygium treatment.

## Supplementary information

**Additional file 1.** The STR verification of HCEs.

**Additional file 2.** Primary culture of pterygium. (a) Primary cells were cultured by tablet culture. (b, c) EMT markers validation with qRT-PCR and immunofluorescence (*p < 0.05, **p < 0.01, ***p < 0.001).

**Additional file 3.** The amplification efficiency of primers. (a-f). The amplification efficiency of primers for miR-199a-3p, miR-199a-5p, U6, DUSP5, MAP3K11 and GAPDH respectively. And the amplification efficiency was performed with amplification curve, melt curve, melt peak and standard curve.

## Data Availability

The datasets used and/or analyzed during the current study are available from the corresponding author on reasonable request.

## Abbreviations

3′-UTR: 3′-untranslated regions
DAPI: 4′,6-diamidino-2-phenylindole
BSA: Bovine serum albumin
CDS: Coding sequence
DNM: Dynamin
ECL: Electrochemical luminescence
EGF: Epidermal growth factor
EMT: Epithelial–mesenchymal transition
FC: Fold change
FISH: Fluorescence in situ hybridization
FITC: Fluorescein isothiocyanate
HCE: Human conjunctival epithelial cell
H-DMEM: High glucose Dulbecco’s modified Eagle’s medium
PBS: Phosphate buffered saline
PMSF: Phenylmethanesulfonyl fluoride
PVDF: Polyvinylidene fluoride
qRT-PCR: Quantitative real-time polymerase chain reaction
shRNA: Short hairpin RNA
STR: Short tandem repeats
TBS-T: Tris buffered saline tween
TGF-β: Transforming growth factor β

## References

[1] Lucas RM, McMichael AJ, Armstrong BK, Smith WT (2008). Estimating the global disease burden due to ultraviolet radiation exposure. Int J Epidemiol.

[2] Lan W, Chen S, Tong L (2015). MicroRNA-215 regulates fibroblast function: insights from a human fibrotic disease. Cell Cycle.

[3] Todani A, Melki SA (2009). Pterygium: current concepts in pathogenesis and treatment. Int Ophthalmol Clin.

[4] Kocamis O, Bilgec M (2014). Evaluation of the recurrence rate for pterygium treated with conjunctival autograft. Graefes Arch Clin Exp Ophthalmol.

[5] Di Girolamo N (2010). Signalling pathways activated by ultraviolet radiation: role in ocular and cutaneous health. Curr Pharm Des.

[6] Wu CW, Peng ML, Yeh KT, Tsai YY, Chiang CC, Cheng YW (2016). Inactivation of p53 in pterygium influence miR-200a expression resulting in ZEB1/ZEB2 up-regulation and EMT processing. Exp Eye Res.

[7] Ando R, Kase S, Ohashi T, Dong Z, Fukuhara J, Kanda A, Murata M, Noda K, Kitaichi N, Ishida S (2011). Tissue factor expression in human pterygium. Mol Vis.

[8] Huerva V, March A, Martinez-Alonso M, Muniesa MJ, Sanchez C (2012). Pterygium surgery by means of conjunctival autograft: long term follow-up. Arq Bras Oftalmol.

[9] Gumus K, Karakucuk S, Mirza GE, Akgun H, Arda H, Oner AO (2014). Overexpression of vascular endothelial growth factor receptor 2 in pterygia may have a predictive value for a higher postoperative recurrence rate. Br J Ophthalmol.

[10] Wu M, Wang J, Zhang Q, Wang Y, Niu L, Shao T (2017). Overexpression of low-density lipoprotein receptors stimulated by vascular endothelial growth factor in fibroblasts from pterygium. Biomed Pharmacother.

[11] Yang J, Weinberg RA (2008). Epithelial-mesenchymal transition: at the crossroads of development and tumor metastasis. Dev Cell.

[12] Zeisberg M, Neilson EG (2009). Biomarkers for epithelial-mesenchymal transitions. J Clin Invest.

[13] Sun Z, Ma Y, Chen F, Wang S, Chen B, Shi J (2018). miR-133b and miR-199b knockdown attenuate TGF-beta1-induced epithelial to mesenchymal transition and renal fibrosis by targeting SIRT1 in diabetic nephropathy. Eur J Pharmacol.

[14] Zhang J, Lang Y, Guo L, Pei Y, Hao S, Liang Z, Su G, Shu L, Liu H, Huang C, Xu J (2018). MicroRNA-323a-3p Promotes Pressure Overload-Induced Cardiac Fibrosis by Targeting TIMP3. Cell Physiol Biochem.

[15] Lino Cardenas CL, Henaoui IS, Courcot E, Roderburg C, Cauffiez C, Aubert S, Copin MC, Wallaert B, Glowacki F, Dewaeles E (2013). miR-199a-5p Is upregulated during fibrogenic response to tissue injury and mediates TGFbeta-induced lung fibroblast activation by targeting caveolin-1. PLoS Genet.

[16] Yang ZC, Qu ZH, Yi MJ, Shan YC, Ran N, Xu L, Liu XJ: MiR-448–5p inhibits TGF-beta1-induced epithelial-mesenchymal transition and pulmonary fibrosis by targeting Six1 in asthma. *J Cell Physiol* 2018. 10.1002/jcp.27540 30362537

[17] Loboda A, Sobczak M, Jozkowicz A, Dulak J (2016). TGF-beta1/Smads and miR-21 in Renal Fibrosis and Inflammation. Mediators Inflamm.

[18] Di Girolamo N, Wakefield D, Coroneo MT (2006). UVB-mediated induction of cytokines and growth factors in pterygium epithelial cells involves cell surface receptors and intracellular signaling. Invest Ophthalmol Vis Sci.

[19] Imaizumi T, Kurosaka D, Tanaka U, Sakai D, Fukuda K, Sanbe A (2019). Topical administration of a ROCK inhibitor prevents anterior subcapsular cataract induced by UV-B irradiation. Exp Eye Res.

[20] Kria L, Ohira A, Amemiya T (1996). Immunohistochemical localization of basic fibroblast growth factor, platelet derived growth factor, transforming growth factor-beta and tumor necrosis factor-alpha in the pterygium. Acta Histochem.

[21] Nuwormegbe SA, Sohn JH, Kim SW (2017). A PPAR-Gamma Agonist Rosiglitazone Suppresses Fibrotic Response in Human Pterygium Fibroblasts by Modulating the p38 MAPK Pathway. Invest Ophthalmol Vis Sci.

[22] Das P, Gokani A, Bagchi K, Bhaduri G, Chaudhuri S, Law S (2015). Limbal epithelial stem-microenvironmental alteration leads to pterygium development. Mol Cell Biochem.

[23] Ghoz N, Britton J, Ross AR, Mohammed I, Hogan E, Said DG, Dua HS (2019). Management of primary pterygium with intra-lesional injection of 5 flurouracil and bevacizumab (Avastin). Eye (Lond).

[24] Chien KH, Chen SJ, Liu JH, Woung LC, Chen JT, Liang CM, Chiou SH, Tsai CY, Cheng CK, Hu CC, Peng CH (2013). Correlation of microRNA-145 levels and clinical severity of pterygia. Ocul Surf.

[25] Chueh-Wei Wu Y-WC, Nan-Yung Hsu, Ken-Tu Yeh, Yi-Yu Tsai, Chun-Chi Chiang, Wei-Ran Wang, Jai-Nien Tung: MiRNA-221 negatively regulated downstream p27Kip1 gene expression involvement in pterygium pathogenesis. *Mol Vis *2014. PMC4105113 25053875

[26] Lee JH, Jung SA, Kwon YA, Chung JL, Kim US (2016). Expression of microRNAs in fibroblast of pterygium. Int J Ophthalmol.

[27] Engelsvold DH, Utheim TP, Olstad OK, Gonzalez P, Eidet JR, Lyberg T, Troseid AM, Dartt DA, Raeder S (2013). miRNA and mRNA expression profiling identifies members of the miR-200 family as potential regulators of epithelial-mesenchymal transition in pterygium. Exp Eye Res.

[28] Cui YH, Li HY, Gao ZX, Liang N, Ma SS, Meng FJ, Li ZJ, Pan HW (2016). Regulation of Apoptosis by miR-122 in Pterygium via Targeting Bcl-w. Invest Ophthalmol Vis Sci.

[29] Bartel DP (2004). MicroRNAs: genomics, biogenesis, mechanism, and function. Cell.

[30] Bartel DP (2009). MicroRNAs: target recognition and regulatory functions. Cell.

[31] Chen BF, Suen YK, Gu S, Li L, Chan WY (2014). A miR-199a/miR-214 self-regulatory network via PSMD10, TP53 and DNMT1 in testicular germ cell tumor. Sci Rep.

[32] Li Y, Wang D, Li X, Shao Y, He Y, Yu H, Ma Z (2019). MiR-199a-5p suppresses non-small cell lung cancer via targeting MAP3K11. J Cancer.

[33] Koshizuka K, Hanazawa T, Kikkawa N, Arai T, Okato A, Kurozumi A, Kato M, Katada K, Okamoto Y, Seki N (2017). Regulation of ITGA3 by the anti-tumor miR-199 family inhibits cancer cell migration and invasion in head and neck cancer. Cancer Sci.

[34] Murakami Y, Toyoda H, Tanaka M, Kuroda M, Harada Y, Matsuda F, Tajima A, Kosaka N, Ochiya T, Shimotohno K (2011). The progression of liver fibrosis is related with overexpression of the miR-199 and 200 families. PLoS ONE.

[35] Ebrahimpour AT, Shrestha S, Bonnen MD, Eissa NTT, Raghu G, Ghebre YT (2018). Nicotine modulates growth factors and microRNA to promote inflammatory and fibrotic processes. J Pharmacol Exp Ther.

[36] Syed V (2016). TGF-beta Signaling in Cancer. J Cell Biochem.

[37] Murillo-Garzon V, Gorrono-Etxebarria I, Akerfelt M, Puustinen MC, Sistonen L, Nees M, Carton J, Waxman J, Kypta RM (2018). Frizzled-8 integrates Wnt-11 and transforming growth factor-beta signaling in prostate cancer. Nat Commun.

[38] Chen XH, Liu ZC, Zhang G, Wei W, Wang XX, Wang H, Ke HP, Zhang F, Wang HS, Cai SH, Du J (2015). TGF-beta and EGF induced HLA-I downregulation is associated with epithelial-mesenchymal transition (EMT) through upregulation of snail in prostate cancer cells. Mol Immunol.

[39] Aranda JF, Canfran-Duque A, Goedeke L, Suarez Y, Fernandez-Hernando C (2015). The miR-199-dynamin regulatory axis controls receptor-mediated endocytosis. J Cell Sci.

[40] Chen T, Margariti A, Kelaini S, Cochrane A, Guha ST, Hu Y, Stitt AW, Zhang L, Xu Q (2015). MicroRNA-199b Modulates Vascular Cell Fate During iPS Cell Differentiation by Targeting the Notch Ligand Jagged1 and Enhancing VEGF Signaling. Stem Cells.

[41] Xu J, Lamouille S, Derynck R (2009). TGF-beta-induced epithelial to mesenchymal transition. Cell Res.

[42] Antoon JW, Nitzchke AM, Martin EC, Rhodes LV, Nam S, Wadsworth S, Salvo VA, Elliott S, Collins-Burow B, Nephew KP, Burow ME (2013). Inhibition of p38 mitogen-activated protein kinase alters microRNA expression and reverses epithelial-to-mesenchymal transition. Int J Oncol.

[43] Zwerina J, Hayer S, Redlich K, Bobacz K, Kollias G, Smolen JS, Schett G (2006). Activation of p38 MAPK is a key step in tumor necrosis factor-mediated inflammatory bone destruction. Arthritis Rheum.

[44] Chen Z, Yu W, Zhou Q, Zhang J, Jiang H, Hao D, Wang J, Zhou Z, He C, Xiao Z (2019). A Novel lncRNA IHS promotes tumor proliferation and metastasis in HCC by regulating the ERK- and AKT/GSK-3beta-Signaling pathways. Mol Ther Nucleic Acids.

[45] Du M, Zhuang Y, Tan P, Yu Z, Zhang X, Wang A (2020). microRNA-95 knockdown inhibits epithelial-mesenchymal transition and cancer stem cell phenotype in gastric cancer cells through MAPK pathway by upregulating DUSP5. J Cell Physiol.

[46] Lee HS, Hwang CY, Shin SY, Kwon KS, Cho KH (2014). MLK3 is part of a feedback mechanism that regulates different cellular responses to reactive oxygen species. Sci Signal.

[47] Wang Z, Reinach PS, Zhang F, Vellonen KS, Urtti A, Turner H, Wolosin JM (2010). DUSP5 and DUSP6 modulate corneal epithelial cell proliferation. Mol Vis.

