# Supplementary figures and images for: MiR-199a-3p/5p participated in TGF-β and EGF induced EMT by targeting DUSP5/MAP3K11 in pterygium
Source: J Transl Med. 2020 Sep 1;18:332. doi: 10.1186/s12967-020-02499-2 (PMC7461358; doi:10.1186/s12967-020-02499-2)

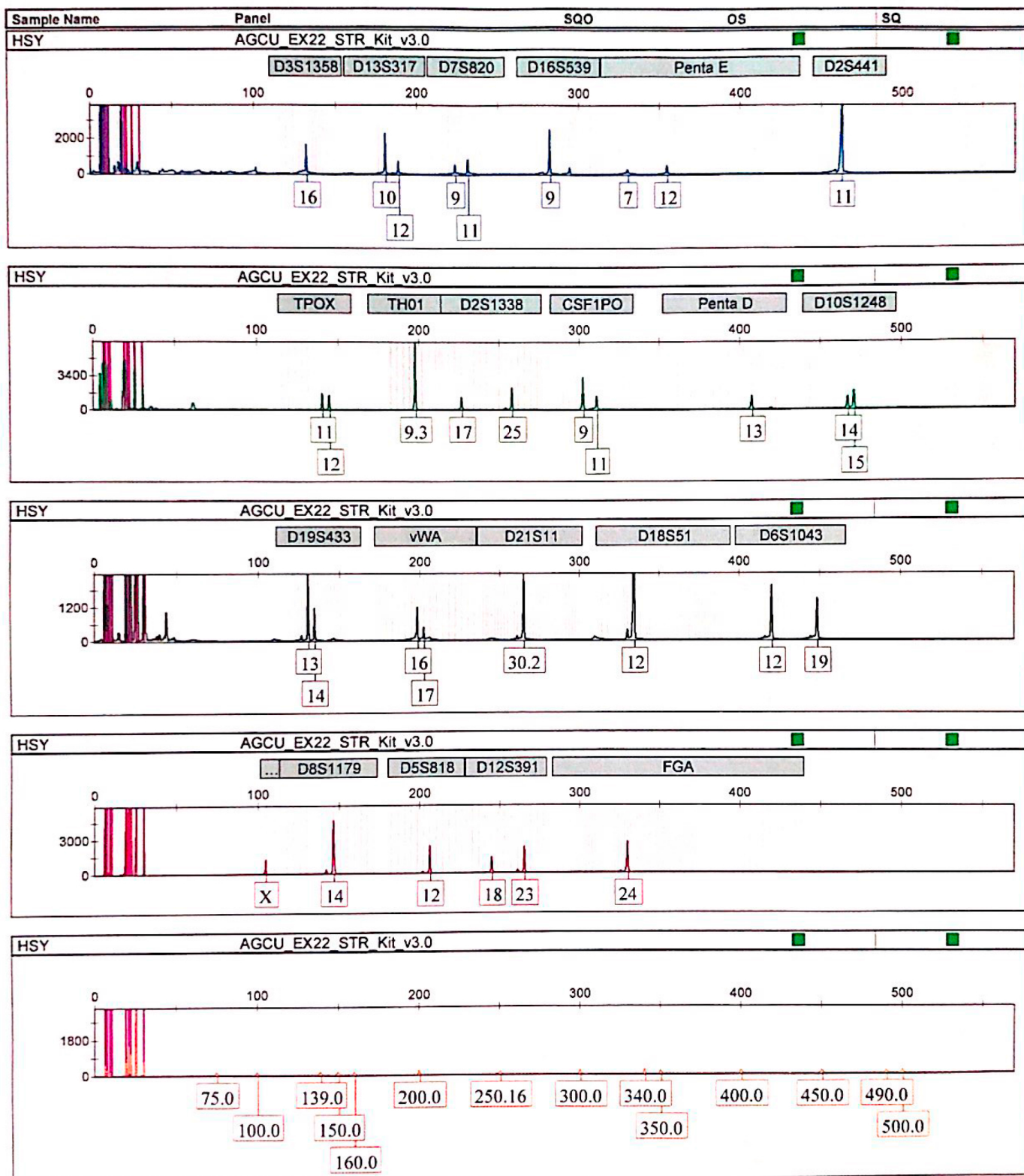

Supplement: Supplementary file 1 — Additional file 1. The STR verification of HCEs. [file 12967_2020_2499_MOESM1_ESM.pdf]

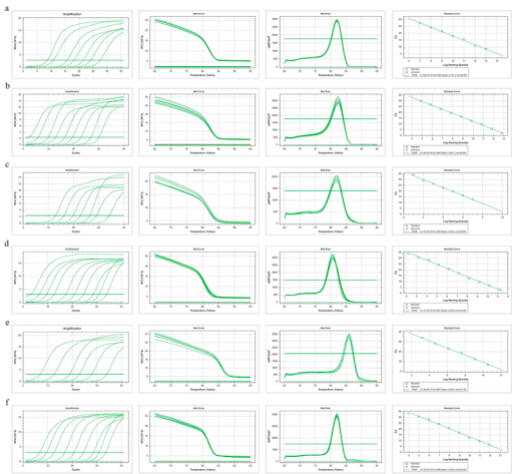

Supplement: Supplementary file 3 — Additional file 3. The amplification efficiency of primers. (a-f). The amplification efficiency of primers for miR-199a-3p, miR-199a-5p, U6, DUSP5, MAP3K11 and GAPDH respectively. And the amplification efficiency was performed with amplification curve, melt curve, melt peak and standard curve. [file 12967_2020_2499_MOESM3_ESM.pdf]
