# Supplementary material for: MiR-199a-3p/5p participated in TGF-β and EGF induced EMT by targeting DUSP5/MAP3K11 in pterygium
Source: J Transl Med. 2020 Sep 1;18:332. doi: 10.1186/s12967-020-02499-2 (PMC7461358; doi:10.1186/s12967-020-02499-2)
Supplement: Supplementary file 2 — Additional file 2. Primary culture of pterygium. (a) Primary cells were cultured by tablet culture. (b, c) EMT markers validation with qRT-PCR and immunofluorescence (*p < 0.05, **p < 0.01, ***p < 0.001). [file 12967_2020_2499_MOESM2_ESM.pdf]

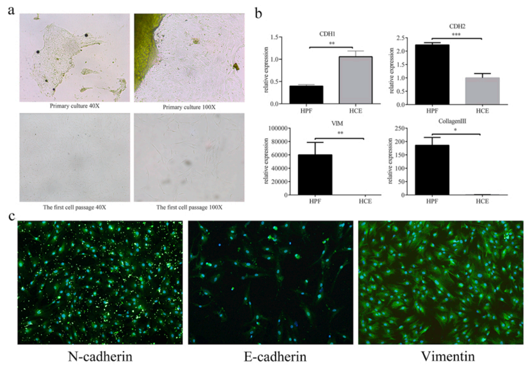
a
Primary culture 40X
Primary culture 100X
The first cell passage 40X
The first cell passage 100X
b
CDH1
**
relative expression
HPF
HCE
CDH2
***
VIM
**
CollagenIII
*
c
N-cadherin
E-cadherin
Vimentin
